# Circulating thyroid hormones and metabolites in children with autism spectrum disorder

**DOI:** 10.3389/fendo.2025.1716586

**Published:** 2026-01-22

**Authors:** Michael Hancock, Rui Zhang, Suzanne J. Brown, Conchita Boyder, Shelby Mullin, Purdey J. Campbell, Scott G. Wilson, Ee Mun Lim, Andrew J.O. Whitehouse, John P. Walsh

**Affiliations:** 1 Department of Endocrinology & Diabetes, Sir Charles Gairdner Hospital, Perth, WA, Australia; 2 PathWest Laboratory Medicine, Queen Elizabeth II (QEII) Medical Centre, Perth, WA, Australia; 3 School of Biomedical Sciences, University of Western Australia, Perth, WA, Australia; 4 Department of Twin Research & Genetic Epidemiology, King’s College, London, United Kingdom; 5 The Kids Research Institute Australia, Perth Children’s Hospital, Perth, WA, Australia; 6 Medical School, University of Western Australia, Perth, WA, Australia

**Keywords:** Australian autism biobank, autism spectrum disorder, liquid chromatography-tandem mass spectrometry (LCMS/MS), thyroid hormones, thyroid metabolites

## Abstract

**Context:**

Thyroid hormones affect neurological development and function, but detailed studies of thyroid hormones and metabolites in autism are lacking.

**Objective:**

To characterize thyroid function and metabolism in autistic children.

**Methodology:**

This cross-sectional study compared 788 autistic children (mean age 7.6 ± 3.9 years, 78% male) with 301 non-autistic children (mean age 7.8 ± 4.0 years, 48% male; comprising 215 (71.4%) non-autistic siblings of participants and 86 (28.6%) unrelated individuals). Plasma TSH, free T4 (FT4) and free T3 (FT3) were measured by automated immunoassay, and total T4, total T3 and thyroid hormone metabolites by customized liquid chromatography-tandem mass spectrometry (LCMS/MS). Regression analyses were adjusted for age and sex.

**Results:**

TSH concentrations were similar in autistic and non-autistic children (median 2.3 vs 2.1 mU/L, P = 0.64). FT4 was significantly lower in autistic children (18.4 vs 18.7 pmol/L, P = 0.0003), as was FT3 (7.0 vs 7.1pmol/L, P<0.0001), with no significant difference in the FT4:FT3 ratio (P = 0.24). Total T4 was lower in autistic children (178 vs 194 nmol/L, P = 0.0026, as was total T3 (2.2 vs 2.4 nmol/L, P = 0.018), with no significant difference in the T4:T3 ratio (P = 0.099). Two metabolites were significantly lower in autistic children: 3,5-T2 (0.010 vs 0.021 nmol/L, P<0.0001) and 3,3’-T2 (0.12 vs 0.16 nmol/L, P<0.0001), whereas T0 levels were higher (1.5 vs 1.1 nmol/L, P = 0.028).

**Conclusions:**

Circulating thyroid hormones and metabolites differ between autistic and non-autistic children, although the observed differences are small. The study demonstrates the utility of LCMS/MS for in-depth characterization of thyroid hormone economy, with potentially wide applications.

## Introduction

Autism spectrum disorder (autism, ASD) refers to a neurodevelopmental disability characterized by qualitative and lifelong differences in social interaction and communication as well as the presence of repetitive and sensory behaviors and interests (1). Most countries with epidemiological data have reported a marked increase in autism diagnoses over recent decades, with current global prevalence estimates of 1.2% to 2%, and a male to female ratio of approximately 3:1 (2, 3). Although the biological pathways contributing to autism remain unclear, the current consensus is for a multifactorial etiology, incorporating a constellation of genetic risk variants interacting with environmental factors (4, 5).

Circulating thyroid hormones have essential roles in brain development and function and are important for childhood development and adult health (6). They have a critical role in in the development of the central nervous system during embryogenesis and early infancy, essential in neurogenesis and oligodendrogenesis, cerebral cortex maturation and myelination (6, 7). The crucial roles of thyroid hormone are demonstrated by the adverse effects of neonatal hypothyroidism on multiple regions and pathways, the most sensitive being cerebral cortex, hippocampus, cerebellum and corticospinal tracts (6).

Under the influence of thyroid stimulating hormone (TSH), the major hormone secreted by the thyroid is thyroxine (T4) which serves largely as a prohormone, being converted to the biologically active hormone triiodothyronine (T3) in peripheral and target tissues. Thyroid hormone biosynthesis, metabolism and regulation are highly complex, far more so than the well described relationships between free T3 (FT3), free T4 (FT4) and TSH. The best established metabolic pathways for thyroid hormones are catalyzed by the iodothyronine deiodinase enzymes D1, D2 and D3, which in a tissue-specific manner convert T4 to T3 and reverse T3, with further deiodination to diiodothyronines, monoiodothyronines and L-thyronine (8, 9). In addition, there are alternate metabolic pathways of decarboxylation to iodothyronamines and oxidative deamination to iodothyroacetic acids. Notably, some metabolites are thought to be biologically active, having been shown to activate nuclear thyroid hormone receptors (8). Tissue concentrations of T3 within the brain are tightly controlled, derived in part from circulating T3, and also from D2-mediated deiodination of T4 (7). In glial cells and certain interneurons, D2 converts T4 to T3, whereas D3 converts T4 and T3 to the inactive metabolites rT3 and T2 respectively (7). The balance between D2 and D3 activity during development is essential to ensure critical neural tissues are exposed to adequate concentrations of T3.

Varying degrees of thyroid dysfunction have been associated with neurodevelopmental and psychiatric phenotypes. Overt and clinically diagnosed thyroid dysfunction are associated with neuropsychological dysfunction, including mood, behavior, emotion, and cognition (10). Variation in thyroid function, even within the normal range, is also associated with impaired mood (10) and cognitive decline in the elderly (11–13), as well as diagnoses of depression (14) and attention deficit hyperactivity disorder (ADHD) (15, 16). In a recent meta-analysis, circulating free T4 concentrations were reported to be reduced in children with neurological disorders (including ADHD, autism and tic disorder) compared with controls, whereas TSH levels were not significantly different (17). In the developing fetus, transplacental passage of maternal thyroxine plays an important role in fetal brain development during the first and second trimester, until the fetal hypothalamo-pituitary-thyroid axis is functionally mature (18). In longitudinal studies, infants exposed *in utero* to either maternal hypothyroidism or thyrotoxicosis are more likely to experience developmental difficulties in motor, social, emotional and language domains (19). Further studies have identified an inverted U-shaped association of higher and lower levels of maternal FT4 during pregnancy (excluding mothers with overt thyroid disease) and reduced cognitive abilities in offspring, as well as lower grey matter and cortex volume (20).

The physiological role of thyroid hormones in brain development makes an association between variation in thyroid function and the biological pathways contributing to autism plausible. Tentative links between thyroid hormone exposure and autism were made in 1970, with suggestions autistic phenotypes may be as a result of delayed diagnosis of thyroid dysfunction (21). Considering this, a small randomized controlled trial with crossover design of 30 clinically euthyroid autistic children was performed, with supraphysiological doses of T3 having no overall benefit on a range of behaviors associated with autism, although there was apparent benefit in a subgroup with lower cognitive abilities (22). These results have not been replicated. Subsequent observational studies were of low quality, with an autistic population different to those in recent clinical practice, and with significant methodological limitations including small sample size and post-mortem tissue analysis (21, 23–25). More recent studies report an increased risk of autism diagnosis in children with a family history of autoimmune thyroid disease (26), and maternal thyroid dysfunction during pregnancy, particularly maternal hypothyroxinemia, has been associated with increased likelihood of autism diagnosis in offspring (15, 27–33). Animal studies have provided evidence that maternal hypothyroxinemia can induce an autism-like phenotype in offspring (34). However, the findings in this area remain inconclusive, and may be affected by misclassification, survival and publication biases (28).

Despite this previous work, there are few detailed studies of thyroid function in autism. A recent case-control study examining thyroid hormones in children with neurological disorders reported altered thyroid parameters, with lower FT4, higher FT3, and lower TSH in children with autism compared with controls (17). To date, there are no data regarding thyroid hormone metabolites in children with autism. We conducted a study of participants in the Australian Autism Biobank, hypothesizing that differences in thyroid hormones and/or metabolites may be present in autistic children compared with non-autistic individuals, and within the ASD group, might be associated with clinical phenotype.

## Methods

A cross-sectional, observational study was performed, examining thyroid hormones and metabolites in autistic children (ASD group) and non-autistic comparators (non-ASD). Data came from the Australian Autism Biobank (AAB), which collected phenotypic data and biological samples from four autism research centers or developmental clinics across Australia (Perth, Brisbane, Melbourne, Sydney), as previously described (35). The ASD group consisted of children with a clinically confirmed diagnosis of autism per the Diagnostic and Statistical Manual of Mental Disorders, 4^th^ and 5^th^ edition (DSM-IV, DSM-5) criteria. The non-ASD group included children with no known developmental differences, comprising non-autistic siblings of autistic participants and unrelated children with no family history of autism.

Participants were excluded if they had a history of thyroid disease, were taking thyroid hormones, antithyroid drugs or medications known to affect thyroid function tests (e.g. lithium, amiodarone, carbamazepine, phenytoin, quetiapine, oral contraceptive and systemic glucocorticoids), had biochemical evidence of clinically significant thyroid dysfunction or results which were outliers (TSH <0.1 or >10 mU/L; FT4, FT3, TSH outside mean ± 4 SD limits).

### Phenotypic data

The AAB contains detailed phenotypic data, including sociodemographic data, family history information, and several clinical assessments completed by trained researchers to gauge behavioral presentation and cognitive abilities.

#### Autistic behavioral characteristics

The Autism Diagnostic Observation Schedule, 2^nd^ Edition (ADOS-2) (36) was administered to the participants in the autism group only. The ADOS-2 is a semi-structured assessment that uses simple activities and questions to elicit and observe communicative and social behaviors relevant to the diagnosis of autism. A range of ADOS-2 modules are available that allows tailoring of activities to the level of verbal language. The ADOS-2 calibrated severity score (range, 1–10 points) was developed to facilitate comparison across different developmentally staged ADOS-2 modules (37). Higher ADOS-2 calibrated severity scores represent greater manifestation of autism behavioral characteristics.

#### Cognitive abilities

One of two measures of cognitive ability was administered to participants, depending on age. Children aged between 2 and 6 years of age were administered the Mullen Scales of Early Learning (MSEL), a standardized developmental assessment involving interactive and play-based tasks (38). Four domains were assessed: fine motor, visual reception, expressive language and receptive language. Children aged above 6 years of age were administered the Wechsler Intelligence Scale for Children 4th edition (WISC-IV) (39), which involves ten structured activities eliciting cognitive abilities in four domains: verbal comprehension, perceptual reasoning, working memory and processing speed. Intellectual disability (ID) was defined as MSEL < 70 or WISC-IV < 70.

#### Functional abilities

The Vineland Adaptive Behavior Scale, 2^nd^ edition (VABS–2) is a parent-report questionnaire that provides a measure of child adaptive behavior (40). The VABS-2 assesses four domains: communication, daily living skills, socialization and motor skills (for children under 6 years of age), which sum together to give an Adaptive Behavior Composite (ABC), standardized around a mean of 100 and standard deviation of 15. A higher ABC indicates greater functional ability. Functional impairment (FI) was defined as VABS-2 ABC < 70.

### Biological sample collection

Venous blood samples were collected from participants under non-fasting conditions by trained pediatric phlebotomists or through hospital/pathology phlebotomy services. Time of blood collection was not standardized, and occurred during office hours (range 0705–1700 h); the median time of collection was 1230 h (inter-quartile range 1149 h, 1330 h). Samples were transported to the University of Queensland’s Institute for Molecular Bioscience for processing, then stored at -80C. Samples were then sent on dry ice to Queen Elizabeth II Medical Centre in Perth for hormone and metabolite analysis. Routine measures of thyroid function were generated from both serum and plasma samples by automated immunoassay using the Roche Cobas e platform (Roche Diagnostics, Australia), measuring FT3, FT4, TSH and thyroid peroxidase antibodies (TPOAb).

Custom developed liquid chromatography-tandem mass spectrometry (LCMS/MS) assays were set up to measure total T4, total T3 and 11 thyroid hormone metabolites (3,3’,5’-triiodothyronine (rT3), 3,3’-diiodothyronine (3,3’-T2), 3,5-diiodothyronine (3,5-T2), 3,5-diiodothyronamine (T2AM), 3 - iodothyronamine (T1AM), 3-iodothyronine (T1), L-thyronine (T0), tetraiodothyroacetic acid (T4A/tetrac), triiodothyroacetic acid (T3A/triac), diiodothyroacetic acid (T2A) and 3-iodothyroacetic acid (T1A)), according to guidelines set out by the Clinical and Laboratory Standards Institute (CLSI C57-ED1) (41). Validation included assessment of limit of detection (LOD), limit of quantification (LOQ), linearity, recovery, matrix effects, specificity, intra-assay repeatability, total analytical repeatability, reference values, robustness, carry-over and sample stability. Full details of assay methodology, validation and performance are provided as Supplementary material, including **Supplementary Tables 1-5**. Calibration standards were made from certified reference material obtained from various suppliers as listed in **Supplementary Table 3**. Acetonitrile protein crush method was used for sample extraction. Fifty microliters of calibrator, quality control and patient samples were spiked with 25 µl of internal standards mixed with 200 µL of 90% acetonitrile 10% methanol solvent solution. After vortexing and centrifugation, 220 µL of supernatant from each sample was collected and dried down in a vacuum concentrator. Dried samples were reconstituted in a solution of 50% of methanol and 50% water. Ten microliters of sample was injected for LCMS for analysis.

LC-MS/MS analysis was performed by a Waters Xevo TQ-XS tandem mass spectrometer coupled with a Waters ACQUITY^®^ I-Class UPLC system with binary pumping capability (Waters Corporation, Milford, Massachusetts, USA) (**Supplementary Table 1**). Experimental setup was programmed by Waters software MassLynx and data was processed with Waters software TargetLynx. Analyte ion detection was performed with positive and negative electrospray ionization in multiple reactions monitoring (MRM) mode (**Supplementary Table 2**). All analytes had acceptable low instrument level of detection for the purpose of the method, acceptable linearity (defined as r2>0.995) over relevant concentration ranges and acceptable level of matrix effects (defined as 100 ± 30%). Assay imprecision was measured by the performance of 3 in-house quality control samples for each analyte; intra-day precision ranged from 2.1-8.0% and inter-day precision from 3.2-14.9% (**Supplementary Table 5**). At the time of establishing the assays (early 2021), no external quality assurance program was available for total T4 and T3 by LCMS/MS; quality control was assured by use of certified reference materials directly to make up standards in methanol with low dilution factors, together with added internal standard in each sample to justify for any sample recovery variance, ion suppression and instrument sensitivity fluctuation.

All 13 analytes were successfully detected on spiked sera (**Supplementary Figure 1**), whereas in participant sera total T4, total T3, L-thyronine (T0), 3,3’,5’-triiodothyronine (reverse T3; rT3), 3,3’-diiodothyronine (3,3’-T2) and 3,5-diiodothyronine (3,5-T2) were detected. The remaining thyroid hormone metabolites were below the limit of detection in all participant samples: 3-iodothyronine (T1), 3-iodothyronamine (T1AM), 3,5-diiodothyronamine (T2AM), 3-iodothyroacetic acid (T1A), diiodothyroacetic acid (T2A), triiodothyroacetic acid (T3A/triac) and tetraiodothyroacetic acid (T4A/tetrac).

### Statistical analysis

Descriptive statistics for continuous variables are given as median and quartiles, and between group comparisons performed using the Mann Whitney U test. Categorical variables are presented as proportions and compared using the Chi-squared or Fisher’s exact test. Skewed variables (all except FT3 and FT4) were then square root or log transformed, as required, before modelling of the mean via regression analysis. Thyroid hormones and metabolites were compared between the ASD and non-ASD groups via linear mixed effects models, which accounted for family relatedness, and adjusted for the covariates age and sex. A generalised linear mixed effects model was used for TPOAb, which was treated as a binary variable using the manufacturer’s recommended reference range of <35 IU/L. The following derived indices of thyroid hormone metabolism and pituitary-thyroid axis function were calculated and compared between ASD and non-ASD: FT4/FT3 ratio, T3/rT3 ratio, FT4xTSH product and TSH index (ln TSH + 0.1345 x FT4) (42, 43).

In the group of autistic children, we assessed associations between thyroid hormones and metabolites and the level of autistic behaviors (using the calibrated severity score of the ADOS-2). Comparisons of analyte levels were made between non-autistic children and (a) autistic children with (ASD+ID) and without (ASDnoID) intellectual disability and (b) autistic children with (ASD+FI) and without (ASDnoFI) functional impairment, as defined above.

Two sensitivity analyses were performed. Firstly, thyroid hormone and metabolite levels were compared between autistic children and each of non-autistic siblings and unrelated controls, as well as between siblings and unrelated controls. Secondly, to examine the potential impact of neurosyndromic diagnoses, such as major chromosomal anomalies and neurological disorders established to be relevant to autism, analyses were performed after excluding children with such diagnoses.

The significance threshold was set at 5% throughout, with false discovery rate adjustment using the Benjamini-Hochberg procedure when multiple comparisons were made in the same analysis. Statistical analysis was performed in the R statistical computing environment, version 4.3.2 (44).

### Ethics approval

The study was conducted in accordance with the Declaration of Helsinki, and approved by the Human Research Ethics Committee of the University of Western Australia (protocol code 2020/ET000102, approved 9th March 2021). Informed consent was obtained from all participants and/or their legal guardians.

## Results

**Figure 1:**
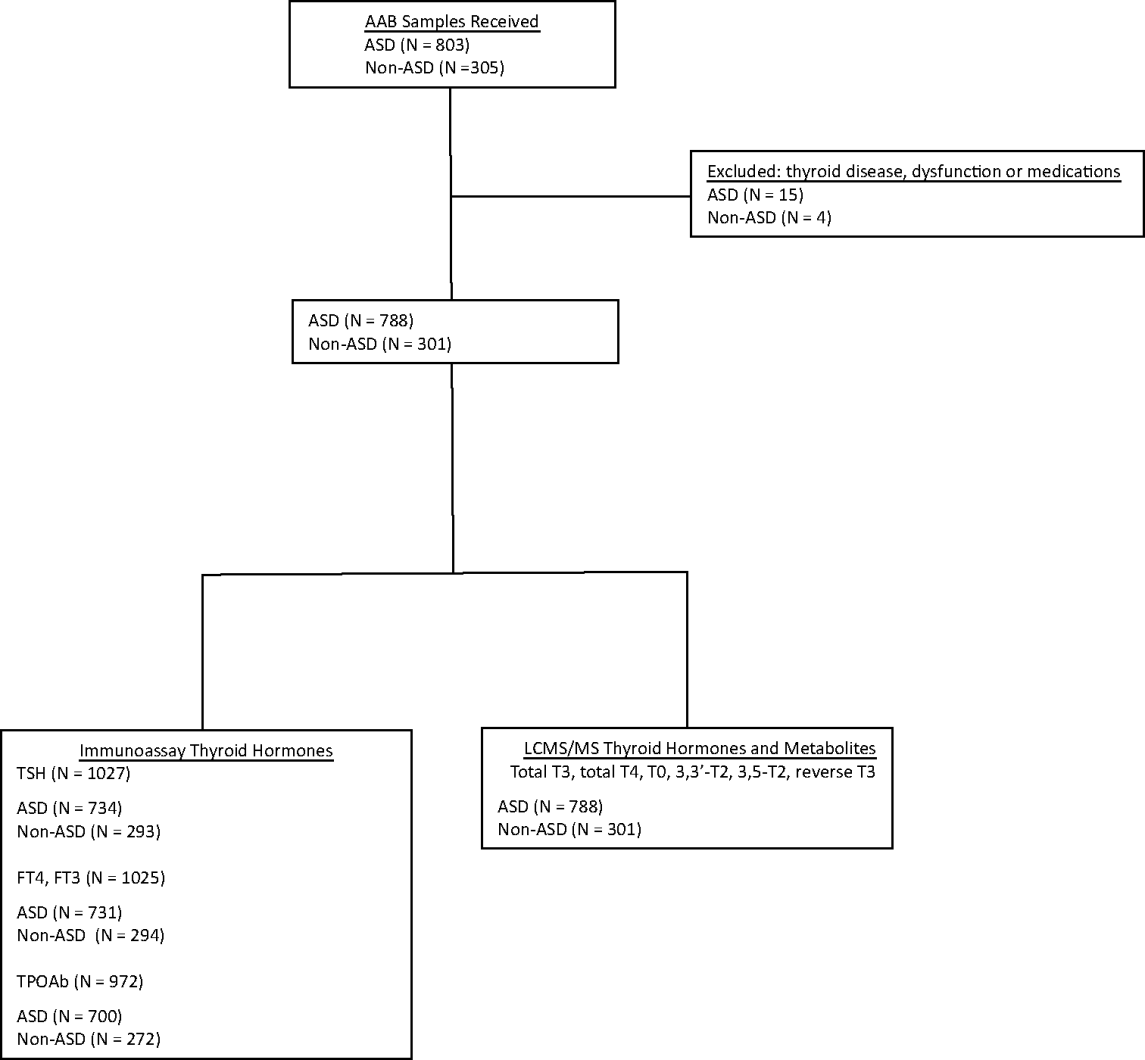
Participant flowchart.

A total of 788 autistic (mean age 7.6 ± 3.9 years, 78% male) and 301 non-autistic children (mean age 7.8 ± 4.0 years, 48% male) were included for analysis, after the exclusion of 19 participants due to a history of thyroid disease, use of thyroid hormones or thyroid-altering medications or biochemical evidence of clinically significant thyroid dysfunction (TSH < 0.1 and > 10 mU/L) (**Figure 1**). Of the 301 non-ASD children, 215 (71.4%) were non-autistic siblings of participants and 86 (28.6%) were unrelated. As expected, the ASD group contained a greater proportion of males and had a higher prevalence of intellectual and functional impairment than the non-ASD group, and autistic children were more likely to be taking prescribed medications (**Table 1**).

**Table 1:** Characteristics of the ASD and non-ASD groups.

Characteristics	ASD	Non-ASD	P-Value
	(N = 788)	(N = 301)	
Age (Years)	7.6 (3.9)	7.8 (4.0)	0.36
Sex (Male)	611 (78%)	144 (48%)	< 0.001
BMI (kg/m^2^)	17.8 (4.2)	17.8 (4.4)	0.82
Neurosyndromic diagnosis	16 (2%)	0 (0%)	0.009
Medication use	193 (24.5%)	23 (7.6%)	< 0.001
Antidepressant/anxiolytic	45 (5.7%)	2 (0.7%)	< 0.001
Antipsychotic/psychotropic	23 (2.9%)	0 (0%)	0.006
Anticonvulsant	31 (3.9%)	1 (0.3%)	0.003
ADHD medication	71 (9.0%)	7 (2.3%)	< 0.001
Melatonin	67 (8.5%)	2 (0.7%)	< 0.001
Anti-asthmatic	31 (3.9%)	10 (3.3%)	0.77
Laxative	9 (1.1%)	2 (0.7%)	0.71
Topical steroid	6 (0.8%)	3 (1.0%)	0.99
Other	27 (3.4%)	7 (2.3%)	0.46
ADOS-2 calibrated severity score (N = 763)	6.7 (1.9)	–	
Mild (1-4)	99 (13%)	–	
Moderate (5-7)	394 (52%)	–	
Severe (8-10)	270 (35%)	–	
Intellectual impairment (N = 919)	237/635 (37%)	3/284 (1%)	< 0.001
Functional impairment (N = 900)	246/599 (41%)	0/301 (0%)	< 0.001

ASD, autistic children; non-ASD, non-autistic children; BMI, body mass index; ADHD, attention deficit hyperactivity disorder; ADOS-2, Autism Diagnostic Observation Schedule, 2^nd^ Edition; N, number.

Values are shown as mean (SD) or n (%).

Thyroid function testing by immunoassay revealed statistically significant differences between autistic and non-autistic children, with lower FT4 (median 18.4 vs 18.7 pmol/L, P = 0.0003) and FT3 (7.0 vs 7.1 pmol/L, P<0.0001) after adjustment for age and sex (**Table 2**). In each case, the magnitude of the difference between groups was small, with median values well within the reference range. TSH, TPOAb and the FT4:FT3 ratio did not differ significantly between groups (P = 0.64, 0.31 and 0.24 respectively).

**Table 2:** TSH, FT4, FT3, FT4:FT3 ratio and TPOAb in ASD and non-ASD groups, measured by immunoassay.

Thyroid-Related Biomarkers	ASD	Non-ASD	β (SE)	P-Value
	(N = 788)	(N = 301)		
TSH (mU/L)	2.3 (1.7, 3.1)	2.1 (1.6, 2.8)	0.011 (0.024)	0.64
FT4 (pmol/L)	18.4 (16.9, 20.0)	18.7 (17.2, 20.4)	-0.63 (0.17)	0.0003
FT3 (pmol/L)	7.0 (6.4, 7.5)	7.1 (6.5, 7.9)	-0.32 (0.06)	< 0.0001
FT4:FT3 ratio	2.7 (2.4, 2.9)	2.7 (2.4, 2.9)	0.033 (0.028)	0.24
TPOAb (kU/L)	14.1 (11.6, 17.0)	14.0 (11.3, 18.8)	-0.42 (0.42)	0.31

Laboratory reference ranges for children (derived from the CALIPER study (62) are as follows:

TSH: 1 to <15 years, 1.1 to 5.0 mU/L; 15 to <19 y, 0.7 to 4.1 mU/L.

FT4: 1 to <19 years, 13 to 21 pmol/L.

FT3: 1 to <14 years, 4.6 to 7.8 pmol/L; 14 to <19 years, 5.0 to 8.2 pmol/L.

ASD, autistic children; non-ASD, non-autistic children; TSH, thyroid stimulating hormone; FT4, free thyroxine; FT3, free triiodothyronine; TPOAb, thyroid peroxidase antibodies; N, number; SE, standard error.

Results are shown as median (IQR).

Total T4 as measured by LCMS/MS was lower in autistic children compared with non-autistic (178 vs 194 nmol/L, P = 0.0026), as was total T3 (2.2 vs. 2.4 nmol/L, P = 0.018), with no significant difference in the T4:T3 ratio (P = 0.099) (**Table 3**). Reverse T3 concentrations did not differ between groups, whereas the T3:rT3 ratio was lower in autistic children compared with non-autistic (2.9 vs 3.1, P = 0.003). Two metabolites had lower concentrations in autistic children compared with non-autistic: 3,5-T2 (0.01 vs 0.021 nmol/L, P<0.0001) and 3,3’-T2 (0.12 vs 0.16 nmol/L, P<0.0001), whereas levels of T0 were higher in ASD than non-ASD (1.5 vs 1.1 nmol/L, P = 0.028).

**Table 3:** Thyroid hormone and metabolite levels in ASD and non-ASD groups, measured by LCMS/MS.

Thyroid-Related Biomarkers	ASD	Non-ASD	β (SE)	P-Value
	(N = 788)	(N = 301)		
Total T4 (nmol/L)	178 (154, 204)	194 (170, 219)	-0.41 (0.13)	0.0026
Total T3 (nmol/L)	2.2 (1.7, 2.7)	2.4 (2.0, 2.9)	-0.075 (0.032)	0.018
Reverse T3 (nmol/L)	0.79 (0.63, 0.91)	0.81 (0.59, 0.98)	0.0045 (0.012)	0.71
3,3'-T2 (nmol/L)	0.12 (0.07, 0.23)	0.16 (0.09, 0.29)	-0.29 (0.050)	<0.0001
3,5-T2 (nmol/L)	0.010 (0.000, 0.025)	0.021 (0.011, 0.033)	-0.85 (0.11)	<0.0001
T0 (nmol/L)	1.5 (0.76, 1.8)	1.1 (0.73, 1.8)	0.072 (0.033)	0.028
T4:T3	81.0 (72.4, 91.6)	77.6 (70.0, 90.5)	3.1 (1.9)	0.099
T3:rT3	2.9 (2.1, 4.1)	3.1 (2.3, 4.5)	-0.12 (0.040)	0.003

ASD, autistic children; non-ASD, non-autistic children; T4, thyroxine; T3, triiodothyronine; 3,3'-T2, 3,3’-diiodothyronine; 3,5-T2, 3,5-diiodothyronine; T0, L-thyronine; N, number; SE, standard error.

Results are shown as median (IQR).

Of the remaining derived indices, the TSH index did not differ significantly between groups (P = 0.07), nor did the FT4xTSH product (P = 0.63).

Within the group of autistic children, there were no significant relationships between the ADOS-2 calibrated severity score, and any of the thyroid hormones and metabolites (**Table 4**).

**Table 4:** Relationships between thyroid hormone and metabolite levels with ADOS-2 calibrated severity score in autistic children.

Thyroid-Related Biomarkers	ADOS-2 calibrated severity score	
	β (SE)	P-Value
TSH (mU/L)	-00002 (0.0068)	0.98
FT4 (pmol/L)	0.089 (0.047)	0.06
FT3 (pmol/L)	0.0095 (0.017)	0.58
Total T4 (nmol/L)	0.031 (0.039)	0.43
Total T3 (nmol/L)	0.01 (0.009)	0.27
Reverse T3 (nmol/L)	0.0015 (0.0032)	0.65
3,3'-T2 (nmol/L)	0.009 (0.015)	0.52
3,5-T2 (nmol/L)	0.014 (0.032)	0.65
T0 (nmol/L)	0.002 (0.01)	0.81

TSH, thyroid stimulating hormone; FT4, free thyroxine; FT3, free triiodothyronine; T4, thyroxine; T3, triiodothyronine; 3,3'-T2, 3,3’-diiodothyronine; 3,5-T2, 3,5-diiodothyronine; T0, L-thyronine; ADOS-2, Autism Diagnostic Observation Schedule, 2^nd^ Edition; SE, standard error.

Higher ADOS-2 scores indicate greater manifestation of autism behavioral characteristics.

**Figure 2:**
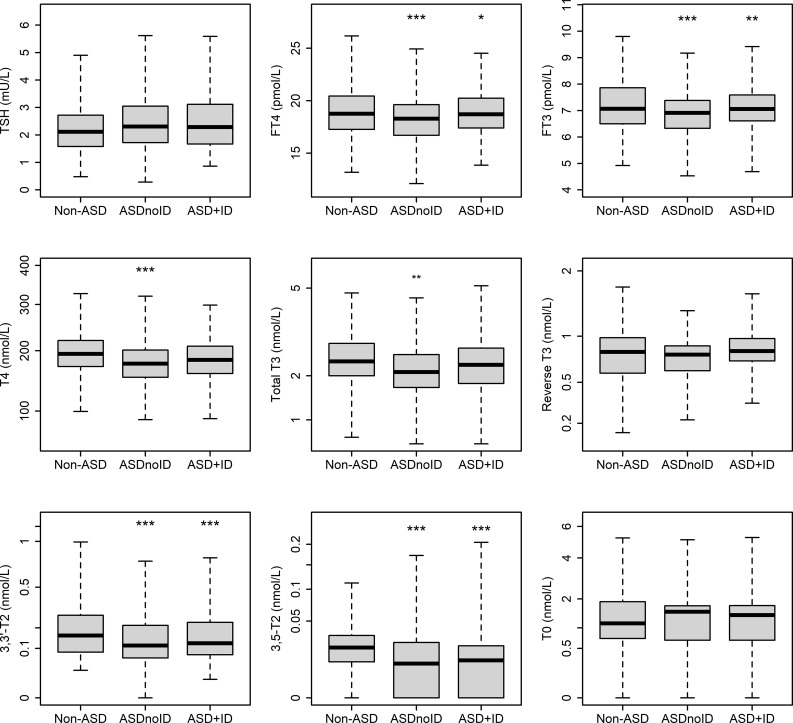
Thyroid hormones and metabolites, measured by immunoassay and LCMS/MS, in autism cases with and without intellectual impairment (ASD+ID and ASDnoID respectively) compared with non-ASD. Data are median, upper/lower quartile and highest/lowest value. *P < 0.05, **P < 0.01, ***P < 0.001.

In subgroups of ASD with and without intellectual disability, results appeared similar to those for the group as a whole (**Figure 2**). FT4, FT3, 3,3’-T2 and 3,5-T2 levels were significantly lower in both subgroups than the non-ASD group. In the subgroup with intellectual disability (37% of cases), total T4 and total T3 were no longer significantly different from non-ASD, and T0 did not differ significantly from non-ASD in either subgroup, probably reflecting reduced statistical power.

**Figure 3:**
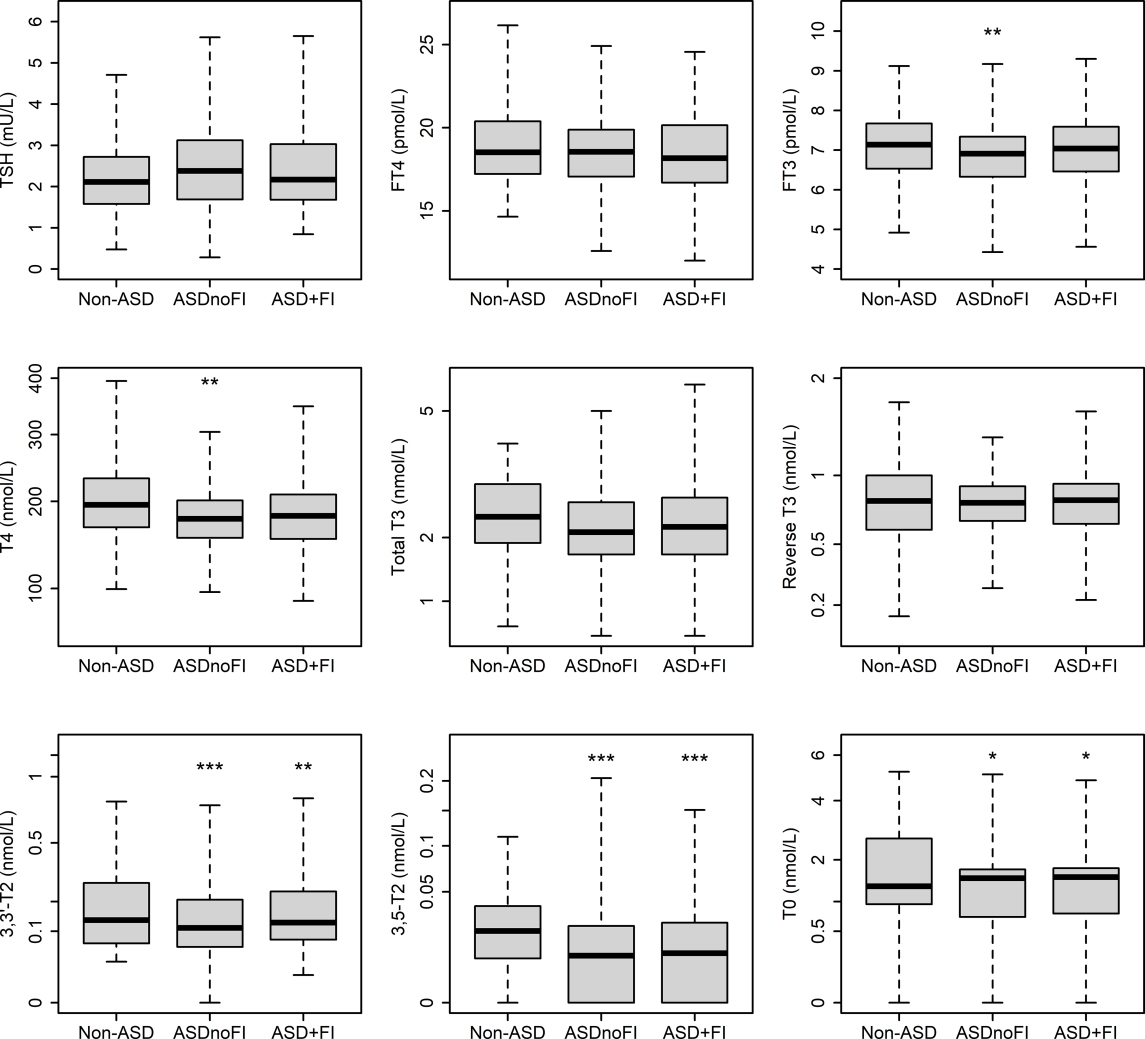
Thyroid hormones and metabolites, measured by immunoassay and LCMS/MS, in autism cases with and without functional impairment (ASD+FI and ASDnoFI respectively) compared with non-ASD. Data are median, upper/lower quartile and highest/lowest value. *P < 0.05, **P < 0.01, ***P < 0.001.

In subgroups of ASD with functional impairment and those without, results appeared broadly similar to those for the cohort as a whole, but differences in FT4 and total T3 were no longer significant compared with non-ASD (**Figure 3**). Levels of 3,3’-T2 and 3,5-T2 levels were significantly lower, and T0 higher in both subgroups than non-ASD (consistent with the entire group), whereas in children with functional impairment (41% of cases), FT3 and total T4 levels did not differ significantly from non-ASD.

In a sensitivity analysis comparing thyroid hormone and metabolite levels between autistic children, non-autistic siblings and unrelated controls, results were similar to those for the primary analysis. Demographic data are shown in **Supplementary Table 6** and results in **Table 5**. For analytes which differed significantly between ASD and non-ASD in the primary analysis, the direction of the effect was the same in the sensitivity analysis, although the magnitude and significance varied with the smaller sample size, and some comparisons were no longer statistically significant. The only analyte which differed significantly between siblings and unrelated controls was T0, being lower in siblings (1.0 vs. 1.3 nmol/L, P = 0.0006), and not significantly different between ASD cases and unrelated controls. In a second sensitivity analysis, results were essentially unchanged after exclusion of 16 autistic children with established neurosyndromic diagnoses relevant to autism, including major chromosomal anomalies and neurological disorders.

**Table 5:** Sensitivity analysis comparing thyroid hormones and metabolites in ASD (N = 788) versus each of non-autistic siblings (N = 215) and unrelated controls (N = 86), and between siblings and unrelated controls.

Thyroid-Related Biomarkers	ASD	Non-Autistic Siblings			Unrelated Controls			Siblings vs. unrelated controls	
	(N = 788)	(N = 201)	β (SE)	P-Value	(N = 86)	β (SE)	P-Value	β (SE)	P-value
TSH (mU/L)	2.3 (1.7, 3.1)	2.1 (1.6, 2.8)	-0.002 (0.026)	0.9400	2.1 (1.6, 2.7)	0.057 (0.044)	0.3249	0.059 (0.048)	0.3249
FT4 (pmol/L)	18.4 (16.9, 20.0)	18.7 (17.2, 20.4)	-0.69 (0.19)	0.0009	18.4 (17.2, 20.3)	-0.42 (0.31)	0.2682	0.26 (0.34)	0.4371
FT3 (pmol/L)	7.0 (6.4, 7.5)	7.1 (6.5, 7.9)	-0.34 (0.066)	1.2E-06	7.2 (6.5, 7.7)	-0.24 (0.11)	0.0475	0.11 (0.12)	0.3702
TPOAb (kU/L)	14.1 (11.6, 17.0)	14.2 (11.7, 18.3)	0.27 (0.49)	0.5830	13.0 (9.8, 21.0)	0.84 (0.66)	0.2040	-0.57 (0.71)	0.4220
FT4:FT3	2.7 (2.4, 2.9)	2.7 (2.4, 3.0)	-0.031 (0.031)	0.3214	2.7 (2.4, 2.9)	-0.040 (0.051)	0.4339	0.009 (0.055)	0.8671
Total T4 (nmol/L)	178 (154, 204)	193 (171, 215)	-0.29 (0.15)	0.0705	196 (167, 230)	-0.76 (0.24)	0.0039	-0.47 (0.26)	0.0705
Total T3 (nmol/L)	2.2 (1.7, 2.7)	2.4 (2.0, 2.9)	-0.082 (0.035)	0.0606	2.4 (1.9, 3.1)	-0.054 (0.055)	0.4873	0.027 (0.061)	0.6510
Reverse T3 (nmol/L)	0.79 (0.63, 0.91)	0.82 (0.61, 0.98)	0.0045 (0.013)	0.9964	0.80 (0.60, 0.99)	0.0046 (0.021)	0.9964	0.00010 (0.023)	0.9964
3,3'-T2 (nmol/L)	0.12 (0.07, 0.23)	0.17 (0.09, 0.32)	-0.30 (0.056)	4.5E-07	0.13 (0.07, 0.28)	-0.26 (0.085)	0.0039	0.044 (0.094)	0.6373
3,5-T2 (nmol/L)	0.010 (0.000, 0.025)	0.021 (0.012, 0.031)	-0.90 (0.12)	6.9E-09	0.021 (0.008, 0.038)	-0.72 (0.18)	7.8E-05	-0.18 (0.20)	0.3631
T0 (nmol/L)	1.5 (0.76, 1.8)	1.0 (0.69, 1.5)	0.13 (0.036)	0.0006	1.3 (0.98, 2.6)	-0.10 (0.058)	0.0732	-0.23 (0.064)	0.0006
T4:T3	81.0 (72.4, 91.6)	77.3 (69.2, 88.3)	-3.1 (1.9)	0.1000	81.4 (70.9, 95.4)	-20.1 (93.0)	0.8291	17.0 (93.0)	0.8551
T3:rT3	2.9 (2.1, 4.1)	3.1 (2.4, 4.5)	0.11 (0.044)	0.0088	3.3 (2.2, 4.5)	0.13 (0.073)	0.0083	-0.011 (0.079)	0.8861

ASD, autistic children; TSH, thyroid stimulating hormone; FT4, free thyroxine; FT3, free triiodothyronine; TPOAb, thyroid peroxidase antibodies; T4, thyroxine; T3, triiodothyronine; 3,3'-T2, 3,3’-diiodothyronine; 3,5-T2, 3,5-diiodothyronine; T0, L-thyronine; N, number; SE, standard error.

Results are shown as median (IQR).

## Discussion

The pathogenesis of autism is poorly understood, but is thought to involve interactions between genetic and environmental factors (4, 5). Thyroid hormones have critical roles in brain development and function (45–47), making it biologically plausible that altered thyroid hormone economy could contribute to the pathogenesis of autism. Thyroid hormone metabolism is complex and tissue-dependent, and although T3 is the most biologically active hormone, metabolites such as 3,5-T2 are also of interest, as they can bind to and activate nuclear thyroid hormone receptors (8), and may have other effects including altered mitochondrial function through non-genomic mechanisms (8, 48, 49).

The aim of this study was to test the hypothesis that alterations in circulating thyroid hormones or metabolites are present in autistic children compared with non-ASD, and might be relevant to autism pathogenesis. Although TSH did not differ significantly between groups, circulating concentrations of total T4 and T3 as well as free T4 and T3 were lower in the ASD than the non-ASD group, accompanied by reduced levels of the thyroid hormone metabolites 3,5-T2 and 3,3’-T2 and higher levels of T0, whereas reverse T3 did not differ significantly between groups. There were no significant relationships between thyroid parameters and autism severity (as assessed by ADOS-2 calibrated severity score). Results appeared consistent across subgroups of ASD with and without intellectual disability or functional impairment, although for some analytes, differences between subgroups and non-ASD were no longer significant, probably reflecting reduced statistical power. Although statistically significant, the magnitude of the differences between ASD and non-ASD groups was small, and likely to be within the bounds of biological variation and uncertainty of measurement. For example, the between-group differences in median FT4 and FT3 (0.3 and 0.1 pmol/L respectively) were less than 2% of the median values, whereas between-subject biological variation for these parameters approximates 8-10% (50). Such small differences are unlikely to be relevant to brain development or function, or to autism pathogenesis.

There are limited previous studies of thyroid function in autism for comparison. In a recent case-control study from China of children with neurological disorders, including 1104 ASD cases and 4801 controls (17), FT4 was significantly lower in autistic children, consistent with our findings. In contrast to our results, however, FT3 was significantly higher in cases (the opposite of our result) and TSH significantly lower (whereas we found no significant difference) and total T4 and T3 not significantly different (whereas we found lower levels of each). In each case, the magnitude of the difference was small (0.6 pmol/L for FT4, 0.2 pmol/L for FT3 and 0.13 mU/L for TSH) and seems unlikely to be clinically relevant. The differences between these results and ours are unexplained; they could reflect differences in participant recruitment (including controls), environmental factors, or methodological differences (for example, medication effects were not accounted for in the previous study).

The mechanism underlying the observed differences in thyroid hormones and metabolites in the present study is uncertain. Circulating concentrations of TSH, T4 and T3 are largely heritable traits, so differences could arise from genetic variants, or from environmental or epigenetic influences (18, 51–53). Individual pituitary-thyroid axis setpoints are thought to be established *in utero*, and can be affected by the intrauterine environment (18, 54) which is also implicated in autism pathogenesis (1). It is possible that the (poorly understood) pathophysiological mechanisms which result in autism also affect hypothalamic development, resulting in altered pituitary-thyroid axis function. Regarding environmental factors, a higher proportion of ASD than non-ASD were taking prescribed medications including anticonvulsants, behavior-modifying and psychotropic drugs. Although we excluded participants on medications with recognized effects on thyroid function tests, subtle effects of other drugs on thyroid hormones and metabolites remain possible. In addition, autistic children often have restricted diets (55), which might alter thyroid hormone metabolism. Mechanistically, a reduction in T4 secretion without alteration in TSH secretion or iodothyronine deiodinase activity could account for the lower circulating levels of the downstream metabolites 3,3’-T2 and 3,5-T2, although it would not account for the higher T0 concentrations.

For the present study, we established customized assays for thyroid hormones and metabolites by LCMS/MS, allowing measurement of total T4, T3, reverse T3, 3,3’-T2, 3,5-T2 and T0 in participants. Assays were also established which detected 7 other thyroid hormone metabolites on spiked sera but were below the limit of detection in participants. There is a limited literature on this methodology in humans for comparison. In a previous study, Jongejan et al. used LCMS/MS to establish a panel of 9 thyroid hormones and metabolites (56). Consistent with our results, they reported undetectable levels of T3A in human sera, but also reported detectable levels of 3-T1 and TA4 which in the present study were below the level of detection. This may reflect differences in LCMS/MS methodology affecting analyte recovery and detection, or possibly developmental effects, since their study was of adults, whereas ours was of children.

Strengths of this study include the large sample size of autistic children, the detailed characterization and expert assessment of participants and the detailed assessment of pituitary-thyroid axis function, including total and free thyroid hormones as well as metabolites by the reference method of LCMS/MS. Our study also has limitations. Timing of blood sampling was not standardized, so results may have been affected by diurnal variation (57) and food intake. We adjusted for age and sex, but not for other factors which are potentially relevant to thyroid function and autism pathogenesis, such as maternal pregnancy history, genetic factors, dietary factors and environmental exposures including endocrine disruptors (58). Arguably, a further limitation is the non-uniform nature of the non-ASD group, which comprised non-autistic siblings of participants and unrelated controls, although this approach has been considered acceptable in previous publications (59–61). In the sensitivity analysis comparing ASD cases with non-autistic sibling and unrelated controls, results were similar to the primary analysis, but it remains possible that comparison with a larger group of unrelated controls would give different results. The only metabolite which differed significantly between non-autistic siblings and unrelated controls was the deiodinated metabolite T0. The basis for this is uncertain, although T0 is thought to be biologically inactive (8).

In conclusion, we report the first study to undertake detailed characterization of pituitary-thyroid axis function and metabolism, including thyroid hormones and metabolites, in a large cohort of autistic children. We found statistically significant differences in circulating thyroid hormones and metabolites in ASD compared with non-ASD, although the magnitude of the differences was small. The study demonstrates the utility of LCMS/MS in detailed characterization of thyroid hormone metabolism, with potential applications in a wide range of clinical and research settings where altered thyroid hormone economy may be present. Further research in independent cohorts would be of interest to confirm our results, to address the discrepant findings between the present study and that of Meng et al. (17). and explore potential mechanisms and implications of altered thyroid hormone economy in autism.

## Data Availability

Thyroid hormone and metabolite data used in this study will be made available by the authors upon request to the Autism CRC/Australian Autism Biobank. Requests to access the datasets should be directed to biobank@autismcrc.com.au.
