# Supplementary material for: Circulating thyroid hormones and metabolites in children with autism spectrum disorder
Source: Front Endocrinol (Lausanne). 2026 Jan 22;16:1716586. doi: 10.3389/fendo.2025.1716586 (PMC12872564; doi:10.3389/fendo.2025.1716586)
Supplement: Supplementary file 1 [file DataSheet1.docx]

**Circulating thyroid hormones and metabolites in children with autism spectrum disorder**

**Supplemental data**

**Methodology and chromatograms for LC-MS/MS Assay of Thyroid Hormone Metabolites**

The method was validated according to guidelines set out by Clinical and Laboratory Standards Institute (CLSI C57-ED1) [1]. Validation included assessment of limit of detection (LOD), limit of quantification (LOQ), linearity, recovery, matrix effects, specificity, intra-assay repeatability, total analytical repeatability, reference values, robustness, carry-over and sample stability.

Acetonitrile protein crush method was used for sample cleanup. 50 µl of calibrator, quality control and patient samples were spiked with 25 µl of internal standards mixed with 200 µL of 90% acetonitrile 10% methanol solvent solution. After vortex for 10 min and sit for 10 min then centrifuge for 10 min at 12,500 rpm, the supernatant of all samples was transferred into separate set of eppendorf tubes and dried down in a vacuum concentrator. Dried samples were reconstituted in a solution constitute of 50% of methanol and 50% water. 10 µL of sample was injected for LCMS for analysis.

LC-MS/MS analysis was performed by a Waters Xevo TQ-XS tandem mass spectrometer coupled with a Waters ACQUITY® I-Class UPLC system with binary pumping capability (Waters Corporation, Milford, Massachusetts, USA). Experimental setup was programmed by Waters software MassLynx and data was processed with Waters software TargetLynx. The LC-MS/MS operation conditions are illustrated in Table 1.

**Table 1**

LC-MS/MS operation conditions.

| Instrumentation | LC: Waters Acquity® I Class UPLC binary pump system | | | | |
| --- | --- | --- | --- | --- | --- |
|  | MS/MS: Waters Xevo TQ-XS triple quadrupole mass spectrometer | | | | |
|  | Ion source: ESI | | | | |
|  | Capillary voltage: +1.8 kV /-2.0kV | | | | |
|  | Drying gas (N_2_) flowrate: 1000L/h | | | | |
|  | Drying gas temperature: 600 ^o^C | | | | |
|  | Collision gas: Argon | | | | |
|  | Detection method: multiple reaction monitoring | | | | |
|  | Injection volume: 10 µl | | | | |
| LC Conditions | Column: Acquity® UPLC BEH C18 column (150 x 2.1 mm) 1.7 µ | | | | |
|  | Column temperature: 40 ^o^C | | | | |
|  | Solvent A: 0.1% Formic acid in LCMS grade water | | | | |
|  | Solvent B: 100 % LCMS grade acetonitrile | | | | |
|  | UPLC Gradient: | Time (min) | Flow Rate (ml/min) | % A | %B |
|  |  | 0.0 | 0.3 | 90 | 10 |
|  |  | 0.5 | 0.3 | 90 | 10 |
|  |  | 10.0 | 0.3 | 40 | 60 |
|  |  | 10.2 | 0.3 | 40 | 60 |
|  |  | 11.5 | 0.3 | 40 | 60 |
|  |  | 11.8 | 0.3 | 5 | 95 |
|  |  | 12.5 | 0.3 | 90 | 10 |
|  |  | 15.0 | 0.3 | 90 | 10 |

Analyte ion detection was performed with positive and negative electrospray ionization in multiple reactions monitoring (MRM) mode. The MRM transitions monitored for quantification and validation are given in Table 2.

**Table 2**

MRM transitions for analytes and internal standards; LC-MS/MS.

| Analyte | MRM Transition (Quantifier) | MRM Transition (Qualifier) | Dwell Time (ms) | Cone Voltage (V) | Collision Energy (V) |
| --- | --- | --- | --- | --- | --- |
| Thyronine (T_0_) | 274.00 > 257.20 | 274.00 > 215.20 | 24 | 30 | (18) / (10) |
| 3 - Iodothyronine (T_1_) | 400.00 > 354.00 | 400.00 > 354.00 | 20 | 30 | 17 |
| 3 - Iodothyronamine (T_1_AM), | 355.98 > 212.10 | 355.98 > 339.10 | 20 | 50 | (15) / (10) |
| 3,3′- Diiodothyronine (3,3′-T_2_) | 525.93 > 381.90 | 525.93 > 478.90 | 80 | 50 | (21) / (20) |
| 3,3′- Diiodothyronine-^13^C_6_ (T_2_-^13^C_6_) | 531.95 > 485.80 |  | 80 | 50 | 20 |
| 3,5 - Diiodothyronine (3,5-T_2_) | 525.95 > 479.90 | 525.95 > 353.00 | 20 | 50 | (29) / (19) |
| 3,5-Diiodothyronamine (T_2_AM) | 481.80 > 468.90 | 481.80 > 338.00 | 20 | 30 | (12) / (14) |
| 3,3′, 5 -Triiodothyronine (T_3_) | 651.70 > 605.60 | 651.70 > 478.80 | 38 | 50 | (20) / (26) |
| 3,3′, 5 -Triiodothyronine-^13^C_6_ (T_3_-^13^C_6_) | 657.60 > 611.60 |  | 38 | 50 | 20 |
| 3,3′, 5′-Triiodothyronine (rT_3_) | 651.83 > 605.80 | 651.83 > 507.80 | 38 | 30 | (23) / (24) |
| 3,3′, 5′-Triiodothyronine-^13^C_6_ (rT_3_-^13^C_6_) | 657.80 > 611.80 |  | 38 | 50 | 25 |
| Thyroxine (T_4_) | 777.50 > 731.50 | 777.50 > 604.50 | 79 | 50 | (25) / (38) |
| Thyroxine-^13^C_6_ (T_4_-^13^C_6_) | 783.50 > 737.50 |  | 79 | 50 | 25 |
| 3-Iodothyroacetic acid (TA_1_) | 368.90 > 126.70 | 324.00 > 126.70 | 52 | 30 | 15 |
| Diiodothyroacetic acid (TA_2_ / Diac), | 450.93 > 126.90 | 324.00 > 126.90 | 52 | 30 | (14) / (25) |
| Triiodothyroacetic acid (TA_3_/Triac) | 576.77 > 126.90 | 576.77 > 449.90 | 52 | 50 | (30) / (19) |
| Tetraiodothyroacetic acid (TA_4_/Tetrac) | 746.65 > 126.90 | 746.65 > 447.90 | 52 | 50 | 26 |

Calibration standards were made from certified reference material (CRM) obtained from various suppliers as listed in Table 3.

**Table 3**

Details of CRM steroid.

| **Analyte** | **Vendor** | **Identifier** | **Concentration** (µmol/L) | **Solvent** |
| --- | --- | --- | --- | --- |
| Thyronine (T_0_) | Toronto research chemicals | 4-MBA-76-4 | 1460.0 | methanol |
| 3 - Iodothyronine (T_1_) | Toronto research chemicals | 2-G5F-126-2 | 5949.7 | methanol |
| 3 - Iodothyronamine (T_1_AM), | Toronto research chemicals | 9-PTR-49-1 | 625.6 | methanol |
| 3,3′- Diiodothyronine (3,3′-T_2_) | IsoSciences | CM001789189-1 | 200.0 | methanol |
| 3,3′- Diiodothyronine-^13^C_6_ (T_2_-^13^C_6_) | IsoSciences | CM00719047-1 | 193.2 | methanol |
| 3,5 - Diiodothyronine (3,5-T_2_) | Toronto research chemicals | 1-KOP-46-1 | 696.6 | methanol |
| 3,5-Diiodothyronamine (T_2_AM) | Toronto research chemicals | 3-JMO-24-1 | 2113.1 | methanol |
| 3,3′, 5 -Triiodothyronine (T_3_) | Cerilliant | FN091912-01 | 15.3 | methanol |
| 3,3′, 5 -Triiodothyronine-^13^C_6_ (T_3_-^13^C_6_) | Cerilliant | FN031913-02 | 3044.5 | methanol |
| 3,3′, 5′-Triiodothyronine (rT_3_) | Cerilliant | FN02051901 | 153.6 | acetonitrile |
| 3,3′, 5′-Triiodothyronine-^13^C_6_ (rT_3_-^13^C_6_) | Cerilliant | FN05121901 | 3805.5 | acetonitrile |
| Thyroxine (T_4_) | Cerilliant | FN031312-01 | 128.7 | methanol |
| Thyroxine-^13^C_6_ (T_4_-^13^C_6_) | Cerilliant | FN102312-02 | 3041.5 | methanol |
| 3-Iodothyroacetic acid (TA_1_) | Toronto research chemicals | 3-MBA-140-1 | 6619.1 | acetonitrile |
| Diiodothyroacetic acid (TA_2_ / Diac), | Toronto research chemicals | 14-BHW-49-1 | 1197.0 | acetonitrile |
| Triiodothyroacetic acid (TA_3_/Triac) | Toronto research chemicals | 14-BHW-118-3 | 1969.7 | acetonitrile |
| Tetraiodothyroacetic acid (TA_4_/Tetrac) | Toronto research chemicals | 6-MBA-68-2 | 1604.6 | acetonitrile |

This assay uses 7 level calibration standards that were made in-house by 1 in 3 serial dilutions of the highest calibration standard which was made by spiking the 13 CRM thyroid metabolites in charcoal stripped serum. Assay calibration ranges and calibration curve fittings are listed in the Table 4.

**Table 4**

Assay calibration range and calibration curve fittings with the lowest (Cal_1) and highest (Cal_7) calibration standards being the lower limit of quantitation (LLOQ) and upper limit of quantitation (ULOQ) respectively.

The instrument limit of detection (LOD) was established, defined as a signal-to-noise (S/N) ratio of 3. All analytes had acceptable low instrument LOD for the purpose of the method. The assay LLOQ were established, defined as the concentration giving a CV% < 20% and shown in the Table 4.

Acceptable linearity was defined as r2>0.995. All analytes had acceptable linearity over a relevant concentration range. All analytes had acceptable level of matrix effects (defined as 100±30%). Assay specificity was defined as the ability to separate relevant isobaric compounds such as T3 and rT3 at baseline level as shown in Figure 1.


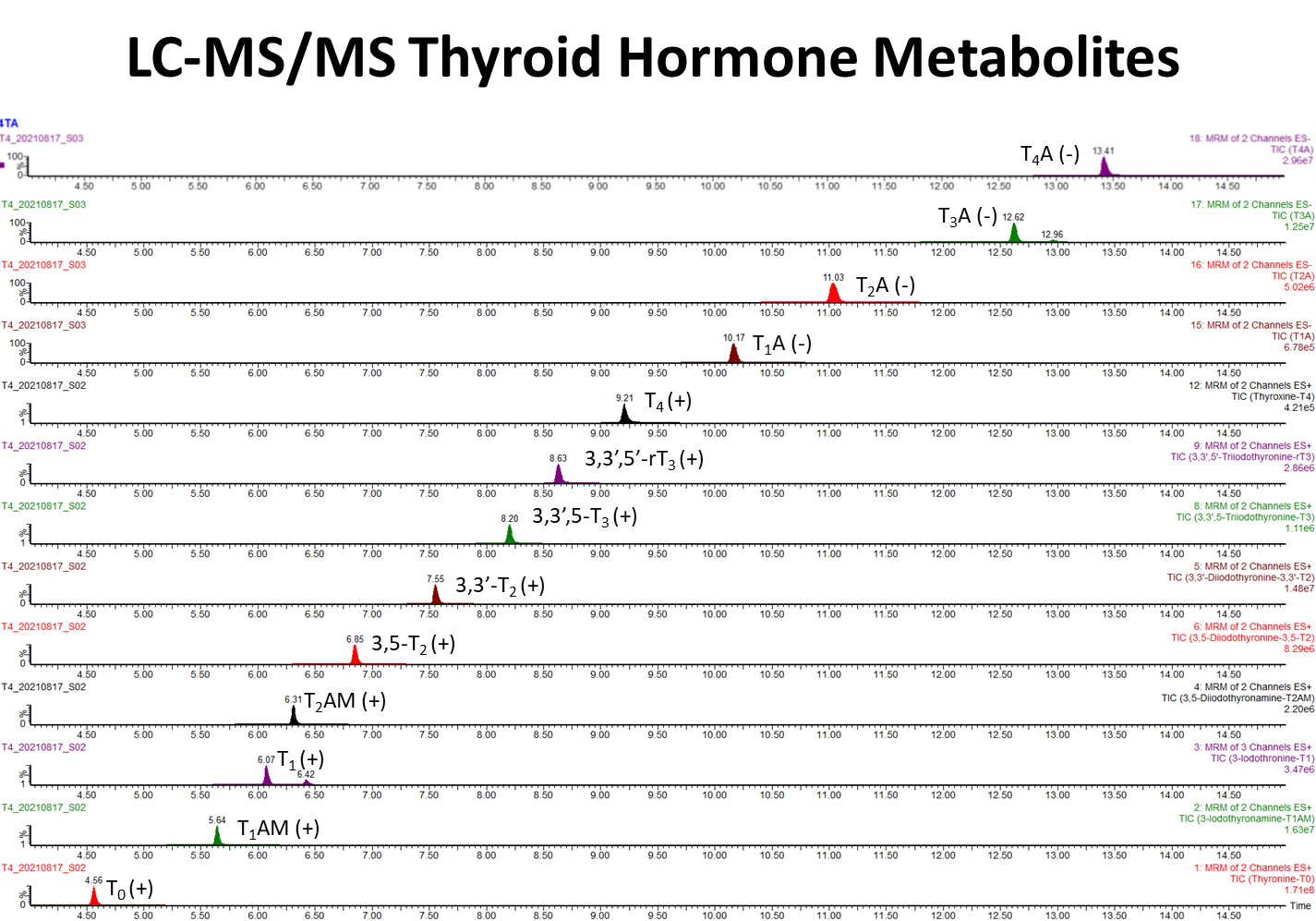


**Figure 1. LC-MS/MS chromatogram of 13 thyroid hormone metabolites.**

The total analytical repeatability was tested prior to routine use. Both intra-day and inter-day assay imprecision are presented in Table 5.

**Table 5**

Intra-day and inter-day assay imprecision were measured by the performance of 3 level of in- house QCs.

The robustness of assay was examined over several month of operation with different lots of solvents, columns, reagents, and other consumables used, as well as instrument sensitivity fluctuation over the time. The method is robust with all the assay criteria maintained.

Carry-over in the LC-system was tested with blank injections after injections of a high analyte concentration samples. Method carry-over was checked by examining a blank injection before and after a high concentration sample. Thyroxines has shown various degree of surface absorption which leads to different levels of carry-over. The surface absorption ability increases with the number of iodine atoms in the thyroxine molecules with T4 showing the highest percentage of carry-over and no carry-over for T0. The assay therefore was programed to run a blank wash injection in between every two samples to eliminate possible carry-over.

Sample stability was studied for extracted samples and they showed a slow degradation over the time in terms of analyte response intensity. Therefore, the assay has adopted an approach that all the extracted samples were analyzed straight after the sample preparation.

**References**

1. Mass Spectrometry for Androgen and Estrogen Measurements in Serum; CLSI C57-ED1, Clinical and Laboratory Standards Institute, 2015.

Table 6: Characteristics of the ASD group, non-autistic siblings and unrelated controls. Values are shown as mean (SD) or n (%).

|  | **ASD** | **Non-ASD** | | **P-Value** | | |
| --- | --- | --- | --- | --- | --- | --- |
|  |  | **Siblings** | **Unrelated** | **ASD v. siblings** | **ASD v. Unrelated** | **Siblings v. Unrelated** |
|  | **(N = 788)** | **(N = 215)** | **(N = 86)** |  |  |  |
| **Age (Years)** | 7.6 (3.9) | 8.2 (4.2) | 6.9 (3.6) | 0.0760 | 0.1650 | 0.0680 |
| **Sex (Male)** | 611 (78%) | 103 (48%) | 41 (48%) | < 0.001 | < 0.001 | 1 |
| **BMI (kg/m^2^)** | 17.8 (4.2) | 18.1 (4.9) | 17.1 (2.9) | 0.3900 | 0.3900 | 0.3900 |
| **Neurosyndromic diagnosis** | 16 (2%) | 0 (0%) | 0 (0%) | 0.0918 | 0.5856 | 1 |
| **Medication use**  **Antidepressant/anxiolytic**  **Antipsychotic/psychotropic**  **Anticonvulsant**  **ADHD medication**  **Melatonin**  **Anti-asthmatic**  **Laxative**  **Topical steroid**  **Other** | 193 (24.5%)  45 (5.7%)  23 (2.9%)  31 (3.9%)  71 (9.0%)  67 (8.5%)  31 (3.9%)  9 (1.1%)  6 (0.8%)  27 (3.4%) | 17 (7.9%)  2 (1.0%)  0 (0%)  1 (0.5%)  4 (1.9%)  2 (0.9%)  9 (4.2%)  2 (0.9%)  3 (1.4%)  5 (2.3%) | 6 (7.0%)  0 (0%)  0 (0%)  0 (0%)  3 (3.5%)  0 (0%)  1 (1.2%)  0 (0%)  0 (0%)  2 (2.3%) | < 0.001  0.0175  0.0229  0.0569  0.0021  < 0.001  1  1  0.9011  1 | < 0.001  0.0653  0.2339  0.1761  0.1844  0.0140  0.5009  1  0.9011  1 | 0.9726  0.9107  1  1  0.6721  0.9107  0.5009  1  0.9011  1 |
| **Intellectual impairment (N = 919)** | 237/635 (37%) | 3/203 (1.5%) | 0/81 (0%) | < 0.001 | < 0.001 | 0.6476 |
| **Functional impairment (N = 900)** | 246/599 (41%) | 0/215 (0%) | 0/86 (0%) | < 0.001 | < 0.001 | 1 |

ASD, autistic children; non-ASD, non-autistic children; BMI, body mass index; ADHD, attention deficit hyperactivity; N, number.
